# Post Procedural Peak Left Atrial Contraction Strain Predicts Recurrence of Arrhythmia after Catheter Ablation of Atrial Fibrillation

**DOI:** 10.1186/s12947-021-00250-5

**Published:** 2021-06-11

**Authors:** Songnan Wen, Manasawee Indrabhinduwat, Peter A. Brady, Cristina Pislaru, Fletcher A. Miller, Naser M. Ammash, Vuyisile T. Nkomo, Ratnasari Padang, Sorin V. Pislaru, Grace Lin

**Affiliations:** 1 Department of Cardiovascular Medicine, Mayo Clinic, 200 First Street SW, MN 55905 Rochester, USA; 2 Department of Cardiology, Bhumibol Adulyadej Hospital, Bangkok, Thailand

**Keywords:** Atrial fibrillation, Ablation, Recurrence, Left atrium function, Strain imaging

## Abstract

**Background:**

Left atrial (LA) function can be impaired by the atrial fibrillation (AF) ablation and might be associated with the risk of recurrence. We sought to determine whether the post-procedural changes in LA function impact the risk of recurrence following AF ablation.

**Methods:**

We retrospectively reviewed patients who underwent AF ablation between 2009 and 2011 and underwent transthoracic echocardiography before ablation, 1-day and 3-month after ablation. Peak left atrial contraction strain (PACS) and left atrial emptying fraction (LAEF) were evaluated during sinus rhythm and compared across the three time points. The primary endpoint was atrial tachyarrhythmia recurrence after ablation.

**Results:**

A total of 144 patients were enrolled (mean age 61 ± 11 years, 77% male, 46% persistent AF). PACS and LAEF initially decreased 1-day following ablation but partially recovered within 3 months in PAF patients, with a similar trend in the PerAF patients. After median 24 months follow-up, 68 (47%) patients had recurrence. Patients with recurrence had higher PACS_1-day_ than that in non-recurrence subjects (-10.9 ± 5.0% vs. -13.4 ± 4.7%, *p* = 0.003). PACS_1-day_ -12% distinguished recurrence cases with a sensitivity of 67.7% and specificity of 60.5%. The Kaplan–Meier curves showed significant difference in 5-year cumulative probability of recurrence between those with PACS ≥ -12% and PACS < -12% (log rank *p* < 0.0001). Multivariate regression showed that PACS_1-day_ was an independent risk factor of arrhythmia recurrence.

**Conclusions:**

Left atrial function deteriorates immediately following AF ablation and partially recovers in 3 months but remains abnormal in the majority of patients. PACS_1-day_ post procedure predicts arrhythmia recurrence at long-term follow-up.

**Supplementary Information:**

The online version contains supplementary material available at 10.1186/s12947-021-00250-5.

## Introduction

Catheter ablation (CA) has become an effective treatment to restore sinus rhythm in atrial fibrillation (AF) patients [1, 2], but AF may recur [3–6]. Left atrial (LA) enlargement is widely regarded as a marker of LA structural remodeling and has been consistently shown to be a powerful predictor of recurrence of AF after cardioversion or ablation [7, 8]. However, AF may also induce significant LA ultrastructural changes, affecting LA myocardial contractility and relaxation even before LA dilatation occurs [9, 10]. The LA strain and LA emptying fraction (LAEF) are measures of LA function and have shown prognostic significance in different pathological conditions including AF [11]. A few studies have suggested that pre-procedural LA strain and LAEF are independent predictors of AF relapse after cardioversion or ablation [12, 13]; however, these measurements may not accurately reflect the performance of LA when obtained during AF. In addition, there is a paucity of data on the effects of catheter ablation on LA function. In the present study, we sought to determine the pattern of LA performance change immediately and short-term after AF ablation and whether such alternation of LA function is related the procedure outcome at long-term follow-up.

## Methods

### Patient population

Patients with symptomatic and drug-refractory AF who underwent first time AF ablation at Mayo Clinic (Rochester, MN) between April 2009 and May 2011 were included. Medical co-morbidities, arrhythmia history, procedural parameters, post-procedure AF treatment, echocardiography and follow-up information were retrieved from the electronic medical record. Patients were excluded if they were < 18 years old, had previous AF ablation or cardiac surgery procedure, valvular AF, congenital heart disease, previous atrioventricular node ablation, permanent pacing or no follow-up data. Paroxysmal AF (PAF) was defined as self-terminating or cardioverted within 7 days of onset. Persistent AF (PerAF) was defined as lasted longer than 7 days [14]. The diagnosis of PAF or PerAF was made by the clinician according to the patient’s medical history and presentation at the time of admission, regardless the actual heart rhythm when the patient was undergoing echocardiography. This study was approved by the Mayo Clinic Institutional Review Board. All patients provided authorization to use their medical records for research purposes.

### Echocardiography study

Transesophageal echocardiography was performed no more than 24 h prior to the procedure to exclude LA thrombus. Transthoracic echocardiography (TTE) was performed before, 1-day and 3-month after ablation. LA volume indexed to body surface area (LAVI), LV ejection fraction (LVEF) by Simpson’s biplane method using manual tracing of digital images, LV diastolic function parameters including mitral E and A velocities, E/A ratio, deceleration time, e’, E/e’ and right ventricular systolic pressure (RVSP) were obtained and measured according to the American Society of Echocardiography guidelines [15].

The atrial cycle can be characterized as three phases: reservoir, conduit and booster, corresponding to LA compliance and distensibility during ventricular systole (atrial filling-reservoir), early diastole with mitral valve opening (conduit), and atrial contraction (booster) during late diastole. Two phases were measured by TTE including booster function (defined as peak LA contraction strain, PACS) and reservoir function (defined as total LA emptying fraction, LAEF), at 3 time points: prior to, 1-day and 3-months after ablation. It was the study protocol that only the PACS and LAEF measurements taken under sinus rhythm would be included for analysis. Left atrial total emptying fraction (LAEF), a measure of LA reservoir function, was calculated from LA volumes as (LA _max vol_ – LA _min vol_)/LA _max vol_ × 100%. PACS was measured by Doppler method as peak negative strain value following the onset of the P wave on the ECG (corresponding to late diastole) and was measured only in the inferior wall of LA because of optimal Doppler alignment in that location. The atrial cycle was used as the reference (zero baseline) point. A sample volume of 2 × 10 mm was placed in the mid-inferior wall in the apical two-chamber view with the image angle aligned as parallel to the region of interest as possible. Data were obtained at a frame rate of > 110 frames per second and sector width adjusted to allow the highest possible frame rate. Measurements from 3 consecutive heart cycles were averaged.

### AF ablation procedure

Patients discontinued amiodarone for 2 months and other antiarrhythmic drugs for 5 half-lives prior to the procedure, according to the standard protocol for AF ablation at Mayo Clinic [16, 17]. Briefly, pulmonary vein (PV) isolation was performed in all patients; additional linear lesions along the LA roof and the left inferior isthmus were added in patients with PerAF. Patients also uniformly underwent cavotricuspid isthmus ablation. Additional ablation targets were non-PV foci in the setting of recurrent spontaneous or induced AF during isoproterenol infusion (5–15 µg/min). Acute procedural endpoints included PVs isolation verified by a circumferential catheter and block of the linear lesions proven by electrophysiological maneuvers, as well as elimination of non-PV foci. If procedural endpoints were achieved but the patient remained in AF, cardioversion was performed at the end of the procedure.

### Follow up

Patients were followed by both Mayo Clinic staff and their cardiologists by means of telephone interview at 30-day post ablation, office visit at 3^rd^ and 12^th^ months after the procedure, and every 12 months thereafter. An initial 3-month blanking period was used when adjudicating arrhythmia recurrence events. At the end of the blanking period, an ECG and 24-h Holter were performed to determine cardiac rhythm status and a TTE was scheduled at the 3-month office visit. After that, patients were instructed to immediately undergo ECG with onset of symptoms suspicious for arrhythmia recurrence. Any atrial tachyarrhythmia including documented AF, atrial flutter, or atrial tachycardia that lasted more than 30 s, and occurred after blanking period, was considered as arrhythmia recurrence.

### Statistical analysis

Continuous variables are presented as means and standard deviations. Comparison of variables between two groups was performed using Student’s t-tests, Fischer exact tests, and Wilcoxon rank sum tests as appropriate. Arrhythmia recurrence risk after ablation was estimated by using the Kaplan–Meier curve and log rank test. Area under the receiver operating characteristic (ROC) curve and univariate logistic regression analysis were used to describe the prognostic value of LA functional parameters for the prediction of recurrence after catheter ablation. Optimal cut-off values were determined by the analysis of the sensitivity and specificity values derived from the ROC curve. We used the Cox proportional hazards regression model to explore and adjust for the effects of baseline characteristics, echocardiographic measurements and other known confounders on the recurrence of atrial arrhythmia. Variables that were statistically significant in univariate regression models (*p* value < 0.1) were included in a multivariate regression model. The PACS and LAEF entered regression model as continuous or category data as appropriate. Intra- and inter-observer variability of echocardiographic measurements was assessed with the Bland–Altman analysis. Statistical analysis was performed using JMP 13.0.0 (SAS Institute Inc.; Cary, NC). A two-sided *p*-value of < 0.05 was considered statistically significant.

## Results

### Clinical characteristics

A total of 144 patients who underwent first time AF ablation were enrolled (mean age 61 ± 11 years; 77% males, mean AF history 6.2 ± 6.1 years). Among them, 78 (54%) were diagnosed as PAF and 66 (46%) as PerAF according to their medical history and clinical presentations. The mean CHA_2_DS_2_-VASc score was 1.8 ± 1.5. Compared with patients in PAF group, patients in PerAF group had more frequent heart failure (26% vs. 3%, *p* < 0.0001) and higher body mass index (32 ± 6 kg/m^2^ vs. 30 ± 5 kg/m^2^, *p* = 0.03). In terms of essential echocardiographic parameters before ablation, patients with PerAF had larger LA volume index (LAVI 43 ± 11 mL/m^2^ vs. 36 ± 9 mL/m^2^, *p* < 0.0001), but lower LV ejection fraction (58 ± 9% vs. 63 ± 5%, *p* = 0.0003) compared with those with PAF. Demographics, clinical characteristics, essential echocardiographic measurements of all patients stratified by the type of AF are shown in Table 1.

**Table 1:** Baseline characteristics of study population

	**Total** **(*n* = 144)**	**Paroxysmal AF** **(*n* = 78)**	**Persistent AF** **(*n* = 66)**	***P* value**
**Demographic Data**				
Age, years	61 ± 11	61 ± 11	60 ± 10	0.95
Men, n (%)	111(77)	55 (71)	56(85)	0.05
Body mass index, kg/m^2^	31 ± 5	30 ± 5	32 ± 6	**0.03**
AF duration, years	6.2 ± 6.1	6.5 ± 6.3	5.8 ± 5.9	0.51
CHA_2_DS_2_-VASc	1.8 ± 1.5	1.8 ± 1.5	1.9 ± 1.5	0.67
**Comorbidity**				
Hypertension, n(%)	74(51)	38(49)	36(55)	0.51
Diabetes, n(%)	15(10)	8(10)	7(11)	0.95
Coronary artery disease, n(%)	22(15)	10(13)	12(18)	0.37
Heart failure, n(%)	19(13)	2(3)	17(26)	** < 0.0001**
Stroke/TIA, n(%)	16(11)	9(12)	7(11)	0.86
**Medication**				
AAD Class I or III, n (%)	70(49)	46(59)	24(34)	**0.01**
β-blocker or CCB, n(%)	95(66)	47(60)	48(73)	**0.04**
**Essential Echo parameters**				
LAVI, mL/m^2^	39 ± 10	36 ± 9	43 ± 11	** < 0.0001**
LVEDD, mm	51 ± 6	50 ± 4	51 ± 5	0.77
LVESD, mm	33 ± 5	32 ± 3	34 ± 6	**0.007**
LVEF,%	60 ± 7	63 ± 5	58 ± 9	**0.0003**
E/A ratio	1.33 ± 0.63	1.30 ± 0.57	1.57 ± 0.91	0.15
RVSP, mmHg	29 ± 6	29 ± 6	29 ± 5	0.64

Values are mean ± SD or number (%)

*AF* atrial fibrillation, *CHA*_*2*_*DS*_*2*_*-VASc* congestive heart failure, hypertension, age ≥ 75 years, diabetes mellitus, prior stroke, transient ischemic attack, or thromboembolism, vascular disease, age 65–74 years, sex category (female), *TIA* transient ischemic attack, *AAD* antiarrhythmic drug, *CCB* calcium channel blocker, *LVESD* left ventricular end-systolic diameter, *LVEDD* left ventricular end-diastolic diameter, *LVEF* left ventricle ejection fraction, *LAVI* left atrium volume index, *RVSP* right ventricular systolic pressure

### LA function before and after AF ablation

**Fig. 1:**
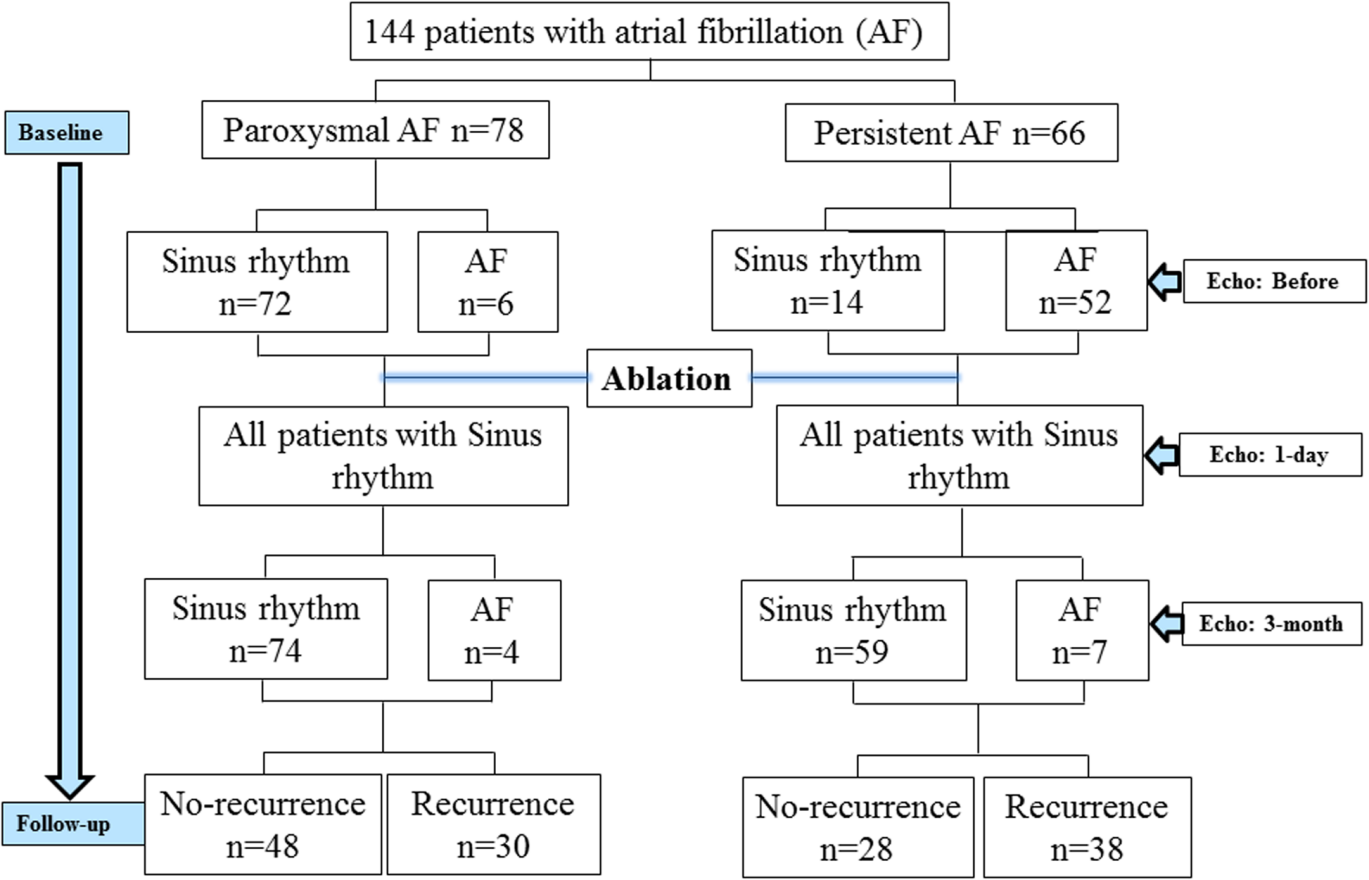
Flow chart of the study protocol

In order to test the variability of strain measurements in our group, we analyzed data of 30 patients. It showed that the intra-observer and inter-observer correlation (GL and MI) was 0.78 (*p* = 0.001) and 0.65 (*p* = 0.03) respectively. The time point when TTE was performed in association with patients’ cardiac rhythm status was illustrated in a flowchart (Fig. 1).

#### ***PACS (PACS***_***before***_***) and LAEF (LAEF***_***before***_***) before ablation***

Eighty-six patients (60%) were in SR at the time of pre-procedural TTE. 72 of them were from the PAF group (72/78, 92%) and 14 were from PerAF group (14/66, 21%). Among the 72 PAF patients, PACS was -17.6 ± 5.5% which was comparable to that of the 14 PerAF patients (-17.3 ± 3.0%, *p* = 0.85) who were in SR at the time of the pre-procedure TTE (Table 2). LAEF_before_ was 44 ± 11% in PAF and 41 ± 11% in PerAF (*p* = 0.50) among patients who were in SR at time of pre-procedural TTE.

**Table 2:** Comparison of left atrium function before, 1-day and 3-month post AF ablation

	**Before ablation**		**1-day after ablation**		**3-month after ablation**		p*	**p#**	**P**^**ǂ**^
	PAF(*n* = 72)	PerAF(*n* = 14)	PAF(*n* = 78)	PerAF(*n* = 66)	PAF(*n* = 74)	PerAF(*n* = 59)			
**PACS, %**	-17.6 ± 5.5	-17.3 ± 3.0	-13.5 ± 4.8	-10.7 ± 4.8	-16.8 ± 5.1	-15.4 ± 4.6	0.77	0.0007	0.09
**LAEF, %**	44 ± 11	41 ± 11	38 ± 9	31 ± 11	41 ± 12	36 ± 10	0.50	** < 0.0001**	**0.009**

^*^Comparison between PAF and PerAF before ablation

^#^ Comparison between PAF and PerAF at 1-day after ablation

ǂ Comparison between PAF and PerAF at 3-month after ablation

*PAF* paroxysmal atrial fibrillation, *PACS *peak atrial contraction strain, *PerAF *persistent atrial fibrillation, *LAEF *left atrial emptying fraction

#### *PACS(PACS*_*1-day*_*)and LAEF(LAEF*_*1-day*_*)1 day after ablation*

All patients remained in SR on day 1 post-ablation. For the entire group, PACS_1-day_ was -12.2 ± 5.0% being higher in PAF patients (-13.5 ± 4.8%) than in PerAF patients (-10.7 ± 4.8%, *p* = 0.0007). Among the 72 PAF patients who were in SR prior to ablation, PACS decreased one day after ablation (-13.8 ± 4.7%) as compared to before ablation (-17.6 ± 5.5%, *p* < 0.0001). Of the 14 PerAF patients who were in SR before ablation, PACS also trended downwards, but was not statistically significant in this small subgroup (-17.3 ± 3.0% vs. -13.1 ± 6.7%, *p* = 0.07; Table 2). On average, the absolute change between PACS_1-day_ and PACS_before_ was 5.89 ± 4.23%.

Similar results were observed for LAEF_1-day_. It was 34 ± 10% in the entire study population, 38 ± 9% in PAF and 31 ± 11% in PerAF patients (*p* < 0.0001). Compared to before ablation, LAEF decreased from 44 ± 11% to 38 ± 9% in PAF patients (*n* = 72, *p* = 0.003) and from 41 ± 11% to 39 ± 13% in PerAF (*n* = 14, *p* = 0.56; Table 2) at day one post ablation. On average, the absolute change between LAEF_1-day_ and LAEF_before_ was 11.1 ± 8.2%.

#### ***PACS (PACS***_***3-month***_***) and LAEF (LAEF***_***3-month***_***) 3 months after ablation***

At the 3-month follow-up TTE, 133 patients remained in SR. Of these, 74 patients were PAF and 59 PerAF group. LA function partially recovered in all groups. PACS_3-month_ and LAEF_3-month_ were -16.8 ± 5.1% (*p* < 0.0001 vs. PACS_1-day_ -13.5 ± 4.8%) and 41 ± 12% (*p* = 0.03 vs. LAEF_1-day_ 38 ± 9%) in PAF patients who remained in SR at 3 months. For PerAF patients in SR at 3 months, PACS_3-month_ and LAEF_3-month_ were -15.4 ± 4.6% (*p* < 0.0001 vs. PACS_1-day_ -10.7 ± 4.8%) and 36 ± 10% (*p* = 0.009 vs. LAEF_1-day_ 31 ± 11%), respectively (Table 2).

**Fig. 2:**
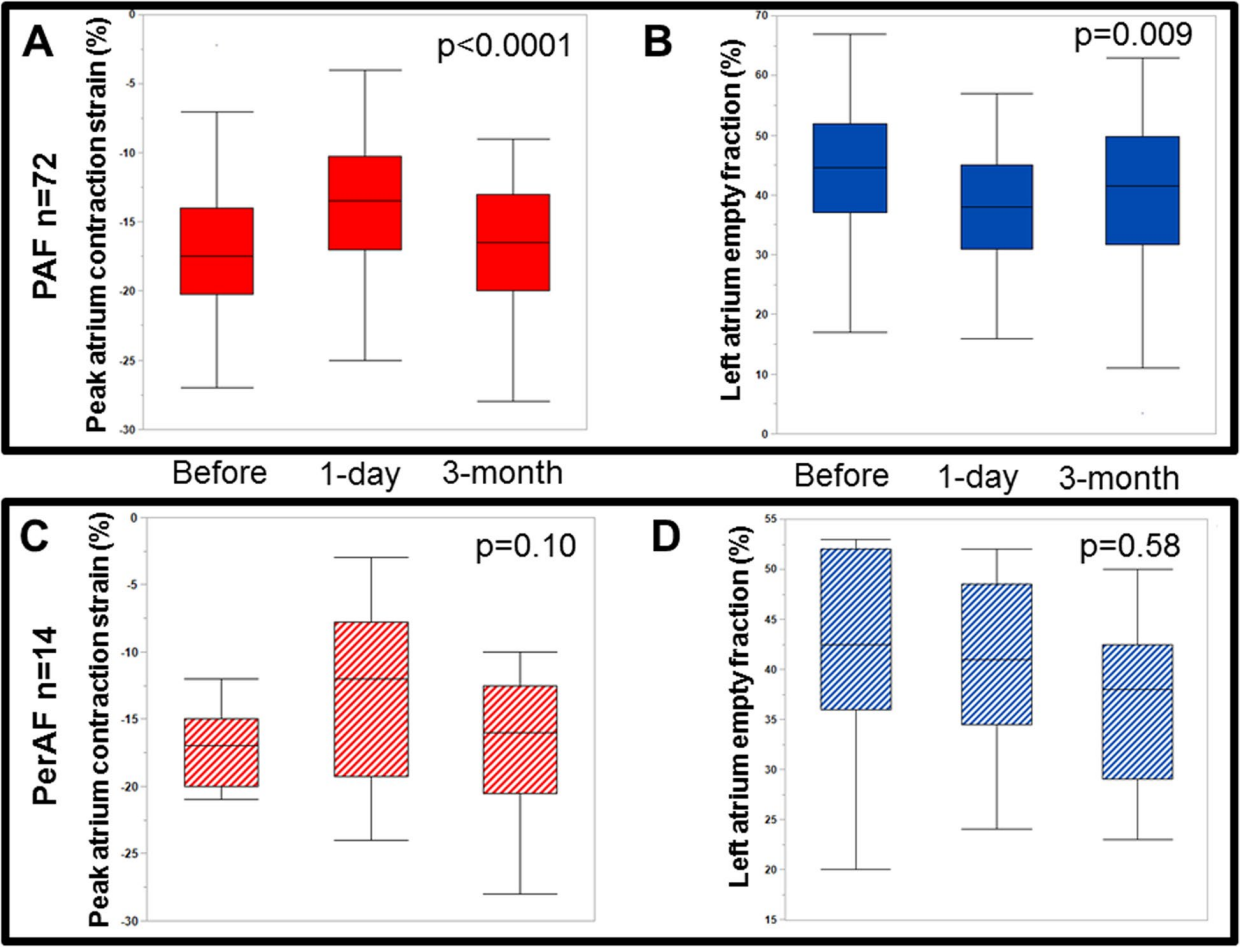
Comparison of peak left atrium contraction strain and left atrium empty fraction across three time points. Data were taken from 72 patients from the PAF group (2A and 2B) and 14 patients from the PerAF group (2C and 2D) who presented in SR before, 1 day and 3 months after ablation. It showed that PACS and LAEF initially decreased 1-day following ablation but partially recovered within 3 months in PAF patients. Similar trend was observed in the PerAF patients. LAEF = left atrial emptying fraction; PACS = peak atrium contraction strain

In 72 PAF patients, PACS and LAEF were available at all three time points. Comparison across these time points showed a significant fluctuation of both PACS (-17.6 ± 5.5% vs. -13.8 ± 4.7% vs. -17.0 ± 5.1%, *p* < 0.0001) and LAEF (44 ± 11% vs. 38 ± 9% vs. 40 ± 12%, *p* = 0.009), where LA function initially decreased and then partially recovered (Fig. 2). A similar trend was observed in the 14 PerAF patients with data available at all 3 time points; but, these changes in PACS did not reach statistical significance (-17.3 ± 3.0% vs. -13.1 ± 6.7% vs.-17.2 ± 5.9%, *p* = 0.10). LAEF (41 ± 11% vs. 39 ± 13% vs.37 ± 8%, *p* = 0.58) did not recover in PerAF patients even in patients who remained in SR at 3 months after ablation.

### Outcome

After median 24 months follow-up, 68 (47%) patients had recurrence of atrial tachyarrhythmia; 30 (38%) in the PAF and 38(58%) in the PerAF group (*p* = 0.03). Comparison of baseline characteristics between patients with and without recurrence is presented in the Table 3.

**Table 3:** Comparison of baseline demographics and clinical characteristics between patients with and without recurrence

	**Comparison**			**Univariate Cox Regression**		
	**No-Recurrence** **(*n* = 76)**	**Recurrence** **(*n* = 68)**	***p* value**	**HR**	**95%Cl**	***P* value**
**Demographic Data**						
Age, years	60 ± 11	61 ± 9	0.63	0.99	0.98–1.02	0.69
Men, n (%)	63 (83)	48(71)	0.06	1.12	0.66–1.90	0.67
Body mass index, kg/m^2^	30 ± 5	31 ± 6	**0.04**	1.04	1.00–1.08	**0.05**
Paroxysmal AF, (%)	48(63)	30(44)	**0.02**	0.62	0.38–1.00	**0.05**
AF duration, years	6.1 ± 6.0	6.2 ± 6.3	0.93	1.00	0.96–1.04	0.99
CHA_2_DS_2_-VASc	1.7 ± 1.5	2.0 ± 1.6	0.26	0.98	0.84–1.14	0.78
**Comorbidity**						
Hypertension, n(%)	35(46)	39(57)	0.12	1.31	0.81–2.13	0.28
Diabetes, n(%)	7(9)	8(12)	0.41	1.05	0.46–2.07	0.90
Coronary artery disease, n(%)	10(13)	12(18)	0.30	1.52	0.77–2.75	0.21
Heart failure, n(%)	9(12)	10(15)	0.40	1.01	0.48–1.88	0.98
Stroke/TIA, n(%)	9(12)	7(10)	0.49	0.68	0.28–1.40	0.32
**Medication**						
AAD Class I or III, n (%)	37(59)	33(49)	0.56	1.03	0.64–1.66	0.91
β-blocker or CCB, n(%)	50(66)	45(66)	0.55	1.43	0.87–2.41	0.16
**Essential Echo parameters**						
LAVI, mL/m^2^	39 ± 10	40 ± 11	0.41	1.00	0.98–1.03	0.55
LVEDD, mm	51 ± 5	50 ± 5	0.75	1.01	0.96–1.07	0.61
LVESD, mm	33 ± 5	33 ± 5	0.69	1.00	0.95–1.05	0.96
LVEF, %	61 ± 8	61 ± 7	0.91	1.00	0.98–1.04	0.63
E/A ratio	1.21 ± 0.52	1.53 ± 0.75	**0.03**	1.39	0.91–2.05	0.13
RVSP, mmHg	29 ± 5	29 ± 6	0.66	1.03	0.98–1.08	0.27

Values are mean ± SD or number (%)

*CI* confidence interval, *HR* hazard ratio; other abbreviations as in Table 1

### LA function and arrhythmia recurrence

**Fig. 3:**
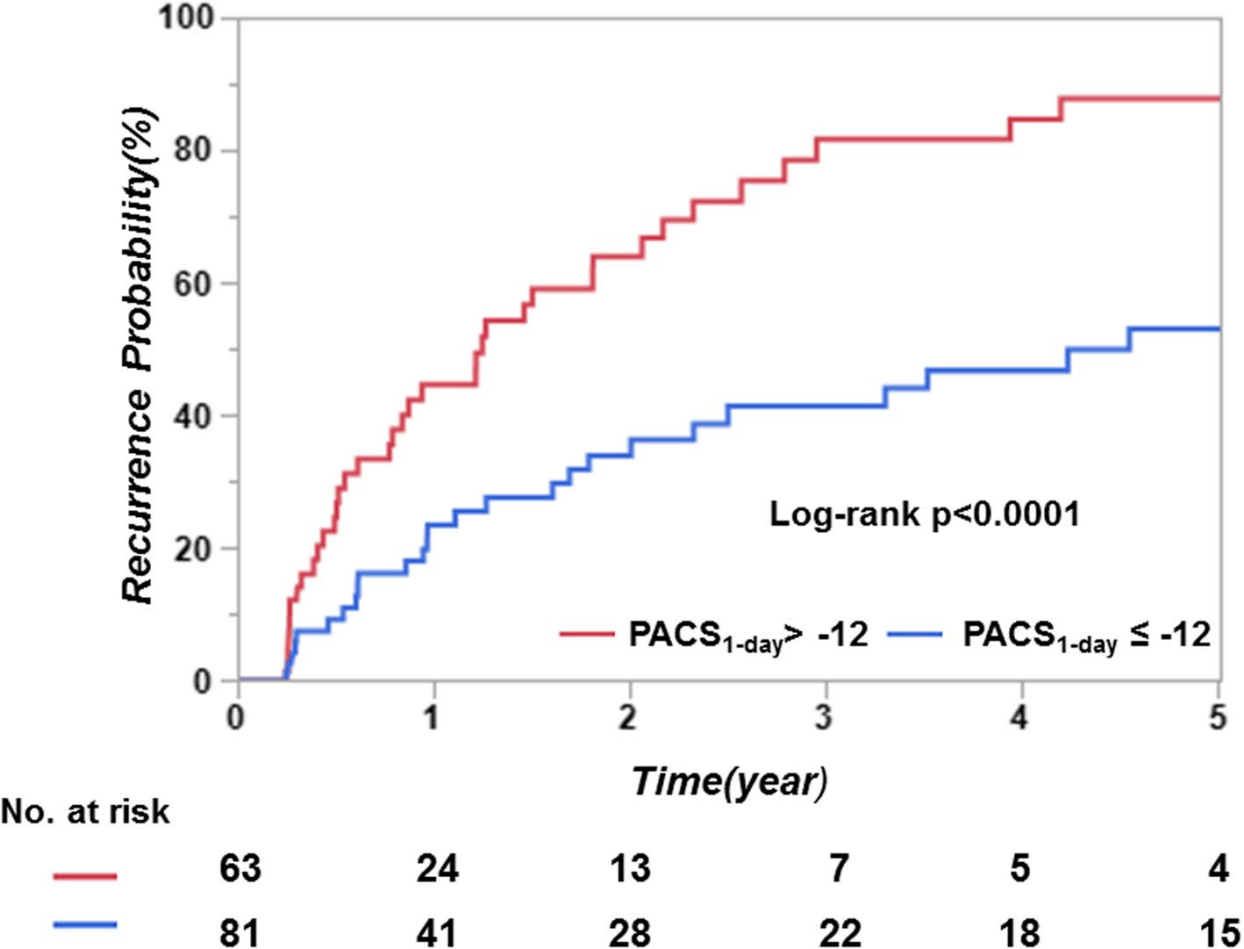
Kaplan–Meier analyses of arrhythmia recurrence according to PACS_1-day_ cut-off value (-12%) in all patients. The analyses showed significant difference in the cumulative probability of arrhythmia recurrence between patients with PACS_1-day_ > -12% and PACS_1-day_ ≤ -12%. PACS = peak atrium contraction strain

We compared PACS and LAEF before ablation, at 1 day and 3 months post ablation while stratifying LA function by arrhythmia recurrence/non-recurrence (Table 4). Univariate Cox regression analysis revealed that only PACS_1-day_ was predictive of recurrence. Baseline PACS and LAEF, LAEF 1 day, and 3 month PACS and LAEF were not different in patients with recurrence vs those without. A ROC was created for the parameters that showed significant difference (*p* < 0.1) in the comparison, to establish the cut-off point with the greatest sensitivity and specificity to predict arrhythmia recurrence. We found that the cut-off value of -12% for the PACS_1-day_ had an area under the curve (AUC) of 0.6574 (*p* = 0.003); with sensitivity of 67.7% and specificity of 60.5% to predict arrhythmia recurrence. The 5-year cumulative recurrence probability was much higher if PACS_1-day_ was ≥ -12% (87.6%, CI 72.2%-95.1%) than for PACS_1-day_ < -12% (52.9%, CI 38.0%-67.3%; log rank *p* < 0.0001, Fig. 3).

**Table 4:** Comparison of left atrium function parameters between patients with and without arrhythmia recurrence and the predictive value to recurrence in univariate Cox regression analysis

	**Comparison of LA function parameters**			**Univariate Cox regression analysis**		
	**No-Recurrence**	**Recurrence**	***p* value**	**HR**	**95%Cl**	***P* value**
**Before Ablation**						
No. of patient in SR	50	36	-	-	-	-
PACS, %	-17.9 ± 6.0	-17.0 ± 3.9	0.40	-	-	-
LAEF, %	44 ± 11	43 ± 11	0.77	-	-	-
**1-day after ablation**						
No. of patient in SR	76	68	-	-	-	
PACS, %	-13.4 ± 4.7	-10.9 ± 5.0	**0.003**	1.11	1.05–1.18	**0.0002**
LAEF, %	36 ± 11	33 ± 10	0.05	0.98	0.96–1.01	0.16
**3-month after ablation**						
No. of patient in SR	76	57	**-**	-	-	-
PACS, %	-16.6 ± 5.0	-15.6 ± 4.8	0.28	-	-	-
LAEF, %	40 ± 12	37 ± 10	0.11	-	-	-

*CI* confidence interval, *HR* hazard ratio, *LA* left atrium, *LAEF* left atrium empty fraction, *PACS* peak atrial contraction strain, *SR* sinus rhythm

### Risk factors of arrhythmia recurrence

A Cox regression model was used to explore the risk factors of arrhythmia recurrence after AF ablation. Potential confounders tested in the univariate analysis were selected based on prior knowledge or expected clinical relevance and from parameters showing significant difference in the Table 3. We found that type of AF and BMI were predictors of recurrence in univariate analysis. These factors were entered into multivariable Cox regression analysis to establish Model 1 together with PACS_1-day_ that was included as continuous data. To further analyze the discrimination power of the cut-off value and the incremental prognostic value of PACS_1-day_, we created Model 2 by including PACS_1-day_ as a binary variant (≥ or < -12%) and Model 3 by including PACS _1-day_ as a category variant (quartiles). The distribution of quartiles of PACS_1-day_ is shown in Supplement Table 1. Comparison of patients’ baseline characteristics stratifying patients by PACS_1-day_ cut-off value and quartiles is reported in Supplement Table 2 and 3.

After adjusting for other confounders including AF type and BMI, PACS_1-day_ (HR 1.11, 95% CI 1.05–1.18, *p* = 0.0003), PACS_1-day_ ≥ -12% (HR 2.53, 95% CI 1.54–4.16, *p* = 0.0003) and PACS_1-day_ quartiles (HR 4.23, 95% CI 2.08–8.75, *P* < 0.0001) were the only independent predictor of arrhythmia recurrence after AF ablation in Model 1, 2 and 3, respectively. Compared with PACS_1-day_ Q4 (> -8%, subjects with the most impaired PACS_1-day_ post procedure), patients in quartiles 1, 2 and 3 had a lower risk of recurrence in the multivariable analysis. Compared to PACS_1-day_ Q1 (≤ -15%), patients in Q4 was associated with nearly 4 times higher recurrence risk (HR 3.82, 95% Cl 1.90–7.68, *p* = 0.0002; Table 5).

**Table 5:** Multivariate Cox regression analysis for predictor of arrhythmia recurrence after ablation

	**Multivariate Cox regression models**			
		**HR**	**95% Cl**	***P* value**
**Model 1:** + BMI, type of AF				
PACS_1-day_ (per unit increase)		1.11	1.05–1.18	**0.0003**
**Model 2:** + BMI, type of AF				
PACS_1-day_ ≥ -12%		2.53	1.54–4.16	**0.0003**
**Model 3A:** + BMI, type of AF				
PACS_1-day_ quartiles all effect		4.23	2.08–8.75	** < 0.0001**
**Model 3B**: + BMI, type of AF				
PACS_1-day_ Q1(reference)		1		
Q2		0.96	0.46–2.01	0.92
Q3		1.85	0.93–3.68	0.08
Q4		3.82	1.90–7.68	**0.0002**
**Model 3C**: + BMI, type of AF				
PACS_1-day_ Q4 (reference)		1		
Q3		0.48	0.26–0.92	**0.03**
Q2		0.25	0.12–0.51	**0.0001**
Q1		0.26	0.13–0.53	**0.0001**

*BMI* body mass index, *CI* confidence interval, *HR* hazards ration, *LA* left atrium, *PACS* peak atrial contraction strain

Univariate analysis demonstrated that the magnitude of difference between PACS_1-day_ and PACS_before_ was associated with increased risk of AF recurrence (HR1.11, 95%CI 1.01–1.22, *p* = 0.03). After adjusting for BMI and AF type, change in pre and post procedure PACS remained significant in multivariate modeling (HR 1.12, 95%CI 1.01–1.23, *p* = 0.03). However, the difference between LAEF_1-day_ and LAEF_before_ did not predict arrhythmia recurrence (HR1.04, 95%CI 0.99 -1.09, *p* = 0.10).

We also observed that PACS_3-month_ and LAEF_3-month_ were predictors of recurrence in univariate analysis in the 133 patients who remained in SR at that time, but this did not reach statistical significance in the multivariable model.

## Discussion

The main findings of our study were as follows: (1) LA booster pump function defined as PACS and reservoir function defined as total LAEF, decline immediately after AF ablation and partially recovers by 3 months post procedure; (2) impaired LA contractile function on the first day after AF ablation is an independent risk factor associated with arrhythmia recurrence at long-term follow-up.

LA function plays a critical role in overall cardiac function, impacting outcomes in patients with heart failure, hypertrophic cardiomyopathy, hypertension and AF [18–21]. Both impaired LA booster (contractile) and reservoir function have been correlated with occurrence of AF and with LA fibrosis assessed by CMR [22–24]. Previous studies have focused on the importance of preserved LA reservoir function in restoration of SR with cardioversion and maintenance of SR following catheter ablation [23, 25]. However, there has been less emphasis on LA booster function. In this study, we observed that impaired peak LA contraction strain immediately following ablation is a risk factor for long term AF recurrence, irrespective of recovery of LA contraction strain at 3 months. We did not observe that reservoir function measured by LAEF at baseline or at 1 day impacted long term maintenance of SR, but this may reflect differences in characterization of LA reservoir function by strain vs. 2D derived LAEF. Nevertheless, our findings suggest that LA contractile function may also play a significant role in occurrence of AF.

Our observation that temporary decline in LA contractile function post-ablation impacts maintenance of SR may have procedural implications as well as implications for patient selection and post ablation management, for example potentially greater value or benefit of prophylactic antiarrhythmic drug therapy and continuation of anticoagulation therapy. Although catheter ablation eliminates trigger activities and/or modifies electrophysiological substrate for AF initiation and maintenances, the procedure induces LA injury which may have longer term deleterious effects on LA structure and function [26]. First, there are direct effects from LA injury: radio-frequency energy damages the atrial myocardium, as evidenced by elevated troponin and tissue edema on imaging studies immediately post procedure, which may lead to fibrosis. Up to 30–35% of the LA wall may be replaced by scar following ablation depending on the extent of ablation and number of procedures [27]. Ablation may also affect autonomic nerve circuits involved in volume modulation of the LA, impairing LA reservoir function [28]. Second, ablation causes LA stunning in > 70% of patients, resulting in decreased contractile and reservoir function [29–32]. The decreased PACS at 1 day and LAEF in our group is consistent with impaired LA function post ablation, either through direct injury or stunning. However, changes in baseline PACS and 1-day after ablation was also associated with increased risk of arrhythmia recurrence suggesting that impairment of LA function post ablation may also be influenced by the severity of LA dysfunction at baseline. If LA dysfunction sustained beyond 3 months as observed in some of our patients, empirically discontinuation of anticoagulant at that the end of blanking period may place the patients at risk of thromboembolism. While our cohort was too small to examine whether differences in ablation techniques (ostial PVI only or more extensive LA linear ablations) impact the severity of LA functional impairment, our findings argue for more thoughtful assessment of the impact of ablation on LA function and methods to identify patients who are most at risk of LA functional impairment and therefore arrhythmia recurrence.

Previous studies investigating LA strain for risk of AF recurrence have assessed LA strain during AF as well as during SR [33]. In patients who are in AF, impaired atrial reservoir function may have the most value in predicting AF recurrence, since booster function is absent and peak LA contraction strain can only be measured during SR [33, 34]. Our findings were therefore limited to patients with PAF and those with PerAF who could be temporarily converted to SR prior to ablation. We could only include a small number of PerAF patients; those patients had larger BMI, LAVI, and more frequent heart failure, and may have had different anatomical, electrophysiological and neuroendocrine profiles as well as degrees of LA remodeling than the patients with PAF [35]. Therefore, peak LA contraction strain following ablation may have different implications for patients with PerAF than PAF. Our observation supports the need for further investigation into the implications of LA booster function for predicting arrhythmia recurrence in both PerAF and PAF after ablation.

Our study is unique and one of few studies that investigated temporal changes in LA function following catheter ablation of AF, including changes in LA booster function. While PACS and LAEF recovered in most patients, LA function remained abnormal, both in comparison to normal reference values and to baseline pre-ablation LA function even for patients remaining in SR. Whether further LA recovery or remodeling can occur beyond 3 months post ablation and affect arrhythmia recurrence risk remains to be determined [36]. In contrast with previous reports we observed that only LA strain one day after ablation, as opposed to baseline or LA strain at 3 months post ablation, is an independent predictor of arrhythmia recurrence [33]. This discrepancy reflects our longer length of follow up and our focus on LA contraction strain rather than LA longitudinal (reservoir) strain. However, our findings are complementary and suggest that both LA contractile and reservoir strain affect the risk of arrhythmia recurrence and should be measured when feasible as part of the pre and post ablation echocardiographic assessment.

### Study limitations

One study limitation is the small sample size, especially of PerAF patients who presented in SR when the baseline TTE was performed. Because of the technology available at the time these patients were studied, we used a tissue Doppler imaging (TDI) method for acquiring LA peak contraction strain. This was measured only for the LA inferior wall, due to the need to optimize the Doppler angle. The inferior wall has the highest deformation value in comparison with the septal and superior segments and has a particularly important role in LA function [37]. However, regional heterogeneity of LA strain has been reported. We acknowledge that the quality of images and accuracy of measurement could be affected by acquisition angle, respiration, etc.; our staff sonographers were trained to maintain the narrowest possible angle of the segments to be measured, with a small sample volume and in expiratory apnea. 2D speckle tracking method has recently become available for online assessment of global peak LA contraction strain, which will make it much easier to incorporate this measurement into clinical practice. Total LAEF was chosen as a measure of reservoir function due to limitations of Doppler-based strain to assess global reservoir function. Future studies with prospective design and 2D speckle tracking strain are warranted to confirm our findings. Finally, documentation of arrhythmia recurrence was not systematic and was driven by patient symptoms and detected by periodic ECG or Holter monitoring. Asymptomatic episodes of atrial tachyarrhythmia may not have been captured, resulting in under-estimation of recurrence.

## Conclusion

Left atrial function significantly deteriorates immediately following AF ablation. It partially recovers in 3 months, but remains abnormal in the majority of patients. Peak left atrial contraction strain on day-one post procedure predicts arrhythmia recurrence at long-term follow-up. This may have further implications for management and risk stratification of patients after catheter ablation of AF.

## Supplementary Information

**Additional file 1**

## Data Availability

The datasets used and/or analyzed during the current study are available from the corresponding author on reasonable request.

## Abbreviations

AF: Atrial fibrillation
CI: Confidence interval
LA: Left atrial
LV: Left ventricle
PerAF: Persistent atrial fibrillation
TTE: Transthoracic echocardiography
AAD: Antiarrhythmic drug
HR: Hazard ratio
LAVI: Left atrial volume index
PAF: Paroxysmal atrial fibrillation
PACS: Peak atrial contraction strain
