# Supplementary material for: Post Procedural Peak Left Atrial Contraction Strain Predicts Recurrence of Arrhythmia after Catheter Ablation of Atrial Fibrillation
Source: Cardiovasc Ultrasound. 2021 Jun 11;19:22. doi: 10.1186/s12947-021-00250-5 (PMC8194218; doi:10.1186/s12947-021-00250-5)
Supplement: Supplementary file 1 — Additional file 1 [file 12947_2021_250_MOESM1_ESM.docx]

|  | **Q1** | **Q2** | **Q3** | **Q4** |
| --- | --- | --- | --- | --- |
|  | **PACS_1-day_≤-15%** | **-15%< PACS_1-day_ ≤-12%** | **-12%< PACS_1-day_ ≤-8%** | **PACS_1-day_ S>-8%** |
| n (%) | 44(31) | 37(26) | 37(26) | 26(18) |

**Supplement Table 1 Distribution of patients by quartiles of PACS_1-day_**

PACS = peak atrial contraction strain

**Supplement Table 2 Comparison of baseline demographics and clinical characteristics stratified by quartiles of PACS_1-day_**

|  | **Total**  **n= 144** | **PACS_1-day_≤-15%**  **n=44** | **-15%< PACS_1-day_ ≤-12%**  **n=37** | **-12%< PACS_1-day_ ≤-8%**  **n=37** | **PACS_1-day_ >-8%**  **n=26** | **P value** |
| --- | --- | --- | --- | --- | --- | --- |
| **Demographic Data**  Age, years | 61±11 | 59±11 | 61±12 | 62±8 | 60±10 | 0.55 |
| Men, n (%) | 111(77) | 33(75) | 27(73) | 28(76) | 23(88) | 0.50 |
| Body mass index ,kg/m^2^ | 31±5 | 30±6 | 30±5 | 31±5 | 31±5 | 0.61 |
| Type of AF |  |  |  |  |  | **0.01** |
| Paroxysmal AF, n(%) | 78(54) | 31(70) | 21(57) | 18(49) | 8(31) |  |
| Persistent AF, n(%) | 66(46) | 13(30) | 16(43) | 19(51) | 18(69) |  |
| AF duration, years | 6.2±6.1 | 6.0±6.6 | 7.6±5.9 | 4.8±4.5 | 6.4±7.4 | 0.28 |
| CHA_2_DS_2_-VASc | 1.8±1.5 | 1.5±1.4 | 2.0±1.6 | 1.9±1.6 | 1.9±1.4 | 0.68 |
| **Comorbidity** |  |  |  |  |  |  |
| Hypertension, n(%) | 74(51) | 22(50) | 18(49) | 18(49) | 16(62) | 0.73 |
| Diabetes, n(%) | 15(10) | 4(9) | 3(8) | 7(19) | 1(4) | 0.23 |
| Coronary artery disease, n(%) | 22(15) | 3(7) | 7(19) | 7(19) | 5(19) | 0.33 |
| Heart failure, n(%) | 19(13) | 4(9) | 1(3) | 5(14) | 9(35) | **0.002** |
| Stroke/TIA, n(%) | 16(11) | 4(9) | 7(19) | 3(8) | 2(8) | 0.38 |
| **Medication** |  |  |  |  |  |  |
| AAD class 1or 3, n (%) | 70(49) | 22(50) | 24(65) | 13(35) | 11(42) | 0.07 |
| β-blocker or CCB, n(%) | 95(66) | 21(48) | 28(76) | 25(68) | 21(81) | **0.01** |
| **Essential Echo parameters** |  |  |  |  |  |  |
| LAVI ,mL/m^2^ | 39±10 | 36±8 | 42±11 | 39±11 | 43±12 | **0.04** |
| LVEDD, mm | 51±6 | 51±4 | 50±5 | 50±4 | 52±5 | 0.18 |
| LVESD, mm | 33±5 | 32±3 | 32±4 | 33±4 | 36±6 | **0.002** |
| LVEF, % | 60±7 | 63±5 | 61±7 | 61±5 | 54±12 | **<0.0001** |
| E/A ratio | 1.33±0.63 | 1.20±0.58 | 1.31±0.57 | 1.38±0.54 | 1.90±0.92 | **0.03** |
| RVSP, mmHg | 29±6 | 29±6 | 29±5 | 28±6 | 29±6 | 0.98 |

Values are mean± SD or number (%).

AF= atrial fibrillation; CHA2DS2-VASc=congestive heart failure, hypertension, age ≥75years, diabetes mellitus, prior stroke, transient ischemic attack, or thromboembolism, vascular disease, age 65-74 years, sex category (female); TIA= transient ischemic attack; AAD=antiarrhythmic drug; CCB=calcium channel blocker; LVESD= left ventricular end-systolic diameter; LVEDD= left ventricular end-diastolic diameter; LVEF=left ventricle ejection fraction; LAVI=left atrium volume index; PACS = peak atrial contraction strain; RVSP= right ventricular systolic pressure.

**Supplement Table 3 Comparison of baseline demographics and clinical characteristics stratified by PACS_1-day_**

|  | **Total**  **n= 144** | **PACS_1-day_<-12**  **n=81** | **PACS_1-day_≥-12**  **n=63** | **P value** |
| --- | --- | --- | --- | --- |
| **Demographic Data** |  |  |  |  |
| Age, years | 61±11 | 60±12 | 61±9 | 0.46 |
| Men, n (%) | 111(77) | 60(74) | 51(81) | 0.33 |
| Body mass index, kg/m^2^ | 31±5 | 30±6 | 31±5 | 0.21 |
| AF of type |  |  |  | **0.006** |
| Paroxysmal AF, n(%) | 78(54) | 52(64) | 26(41) |  |
| Persistent AF, n(%) | 66(46) | 29(36) | 37(59) |  |
| AF duration ,years | 6.2±6.1 | 6.8±6.3 | 5.5±5.8 | 0.21 |
| CHA2DS2-VASc | 1.8±1.5 | 1.8±1.5 | 1.9±1.5 | 0.64 |
| **Comorbidity** |  |  |  |  |
| Hypertension, n(%) | 74(51) | 40(49) | 34(54) | 0.59 |
| Diabetes, n(%) | 15(10) | 7(9) | 8(13) | 0.43 |
| Coronary artery disease, n(%) | 22(15) | 10(12) | 12(19) | 0.27 |
| Heart failure, n(%) | 19(13) | 5(6) | 14(22) | **0.005** |
| Stroke/TIA, n(%) | 16(11) | 11(14) | 5(8) | 0.29 |
| **Medication** |  |  |  |  |
| AAD class 1or 3, n (%) | 70(49) | 46(57) | 24(38) | **0.03** |
| β-blocker or CCB, n(%) | 95(66) | 49(60) | 46(73) | 0.12 |
| **Essential Echo parameters** |  |  |  |  |
| LAVI, mL/m^2^ | 39±10 | 39±10 | 40±11 | 0.34 |
| LVEDD, mm | 51±5 | 50±5 | 51±5 | 0.83 |
| LVESD, mm | 33±5 | 32±4 | 34±5 | **0.03** |
| LVEF, % | 61±8 | 62±6 | 58±9 | **0.002** |
| E/A ratio | 1.33±0.64 | 1.24±0.58 | 1.55±0.71 | 0.05 |
| RVSP, mmHg | 29±6 | 29±6 | 29±6 | 0.91 |

Values are mean± SD or number (%).

AF= atrial fibrillation; CHA2DS2-VASc=congestive heart failure, hypertension, age ≥75years, diabetes mellitus, prior stroke, transient ischemic attack, or thromboembolism, vascular disease, age 65-74 years, sex category (female); TIA= transient ischemic attack; AAD=antiarrhythmic drug; CCB=calcium channel blocker; LVESD= left ventricular end-systolic diameter; LVEDD= left ventricular end-diastolic diameter; LVEF=left ventricle ejection fraction; LAVI=left atrium volume index; PACS = peak atrial contraction strain; RVSP= right ventricular systolic pressure.
